# Editorial: Pancreatic cystic lesions: aiding in the early diagnosis of pancreatic cancer

**DOI:** 10.3389/fgstr.2023.1355275

**Published:** 2024-01-11

**Authors:** Ravi Kumar Sharma, Puneet Chhabra, Surinder Singh Rana

**Affiliations:** ^1^ Department of University Institute of Biotechnology, Chandigarh University, Mohali, Punjab, India; ^2^ Department of Gastroenterology, Calderdale and Huddersfield NHS, Trust, Yorkshire, United Kingdom; ^3^ Department of Gastroenterology, Postgraduate Institute of Medical Education and Research, Chandigarh, India

**Keywords:** pancreatic cystic lesions, mucinous cyst, CEA, cyst fluid analysis, EUS-FNA, KRAS

Pancreatic Cystic Lesions (PCLs), recognized as precursor lesions for pancreatic cancer, represent a diverse group of cystic tumors with varying etiologies and malignant potential (1). Over recent decades, advancements in imaging modalities have led to a significant increase in PCL diagnoses. (2) Currently, most diagnosed PCLs are small, with about 50% of patients being asymptomatic and incidental (3). Despite their small size and asymptomatic nature, these PCLs can potentially transform into pancreatic cancer. The management of PCLs is determined by their etiology and malignant potential. For example, mucinous cysts and solid pseudopapillary tumors (SPTs) necessitate surgical intervention, while inflammatory pseudocysts are typically treated endoscopically. Benign cysts, such as serous cystadenomas (SCA), are monitored through follow-ups (4).

Diagnostic approaches for PCLs have significantly evolved over the last two decades. While cytology has limited sensitivity due to low cellularity and radiology struggles to accurately differentiate PCLs, both prove highly useful in the context of malignant or high-risk PCLs.

Endoscopic Ultrasound (EUS) has emerged as a crucial tool, providing high-quality images of PCLs, pancreatic duct structure, communication with cysts, pancreatic parenchyma, vascularity, and calcification. EUS-Fine Needle Aspiration (FNA) is instrumental in acquiring cyst fluid for further analysis (4). This fluid offers an opportunity to solve the diagnostic puzzle of PCLs through a multimodal analysis involving EUS-FNA, string tests, biochemical assessments, cytological examinations, tumor markers, and molecular analysis. String tests, which can diagnose mucinous cysts immediately after cyst fluid acquisition, have a sensitivity of about 58%-65% (5).

Carcinoembryonic Antigen (CEA) is considered a reliable marker in the differential diagnosis of mucinous and non-mucinous PCLs. Despite controversies over lower versus higher cutoff values compromising its diagnostic importance, CEA remains the most studied and trusted marker of cyst fluid analysis. Studies from 2000 to 2014 showed cutoff ranges >192-800 ng/mL with sensitivity ranges of 48-73% and specificity between 84-98%. (3, 4). However, studies from 2014 onwards showed lower cutoffs from 30-45ng/ML with sensitivity ranges of 85-89% and specificity of 96-98% (P<0.0001). This lower cutoff could be attributed to the small size of PCLs. (5, 6).

Lower cyst fluid glucose levels have been associated with high sensitivity and specificity for the diagnosis of mucinous versus non-mucinous PCLs (7). Although there is less research on this than CEA, meaning CEA remains the superior marker for differentiating mucinous cysts.

Point mutations in KRAS and GNAS have shown sensitivity of 70–100% and specificity of 95–100% for the diagnosis of mucinous cysts. (5, 8). The NGS gene panel of cyst fluid, including point mutations in KRAS, GNAS, BRAF, NRAS, CDKN2A, CTNNB1, SMAD4, TP53, PIK3CA, RNF43, and VHL, as well as loss of heterozygosity and aneuploidy, can provide an accurate diagnosis of PCLs. The VHL point mutation was exclusively noted in SCA, and GNAS was found to be specific for IPMN. (5, 8, 9).

This current Research Topic includes a variety of contributions in the form of original articles, review articles, novel methods, and case studies. Dhani et al. performed a protein-based exosome biomarker test in liquid biopsy samples for the early diagnosis of pre-cancerous lesions, potentially improving patient outcomes. Sheik et al. used a low volume assay for the early diagnosis of pancreatic cancer, demonstrating that as little as 50 µL of cyst fluid is sufficient to diagnose non-mucinous PCLs without malignant potential. This study could be particularly useful in cases where cyst fluid is scarce. Zhang et al. used an artificial intelligence algorithm for the diagnosis of Serous cystic neoplasm and Mucinous cystic neoplasm, proving that it was more effective than conventional radiology. This study showed that AI tools can be useful in early diagnosis of PCLs. Dai el al. showed that fatty acid metabolism subtypes have prognostic and therapeutic relevance in pancreatic cancer. 

The precise differential diagnosis of PCLs necessitates a comprehensive evaluation of all radiological and cytological findings, complemented by an analysis of cyst fluid, which includes Cyst fluid CEA, glucose levels, and molecular markers. This complexity indicates that AI could significantly contribute to future diagnostic processes.

## Author contributions

RKS: Writing – original draft. PC: Writing – review & editing. SSR: Writing – review & editing.
